# Cytotoxic T-lymphocyte-associated protein 4 +49A/G polymorphisms contribute to the risk of type 1 diabetes in children: An updated systematic review and meta-analysis with trial sequential analysis

**DOI:** 10.18632/oncotarget.14457

**Published:** 2017-01-02

**Authors:** Bo Wang, Wei Du, Yutao Jia, Xiaobai Zhang, Guorui Ma

**Affiliations:** ^1^ Department of Paediatrics, The First Affiliated Hospital of Henan University of Science and Technology, Luoyang, Henan, China; ^2^ Department of Medical Laboratory, Luoyang Central Hospital, Luoyang, Henan, China

**Keywords:** type 1 diabetes, cytotoxic T-lymphocyte associated protein 4, polymorphism, risk, meta-analysis

## Abstract

Type 1 diabetes (T1D) is a heritable disease associated with multiple genetic variants. This systematic review and meta-analysis assessed the correlation between cytotoxic T-lymphocyte-associated protein 4(CTLA-4) +49A/G polymorphisms and the risk of T1D in children. The random effects model was used to estimate the related odds ratios (ORs) and 95% confidence intervals (CIs). Trial sequential analysis (TSA) was used to determine whether the currently available evidence was sufficient and conclusive. Our results indicated that *CTLA-4* gene polymorphisms significantly increased the risk of childhood T1D in an allelic model (G vs. A: OR=1.33, 95%CI=1.19-1.48; *I^2^*=44.0% and *P*=0.001for heterogeneity) and a codominant model (GG vs. AA: OR=1.75, 95%CI=1.37-2.24; *I^2^*=57.5% and *P*=0.001for heterogeneity; GA vs. AA: OR=1.26, 95%CI=1.09-1.46; *I^2^*=40.4% and *P*=0.036for heterogeneity). Subgroup analysis results indicated that the ORs were higher in the Asian population (OR_*allelic model*_=1.60, OR_*GG vs. AA*_=2.46 and OR_*GA vs. AA*_=1.58) than the Caucasian population (OR_*allelic model*_==1.24, OR_*GG vs. AA*_=1.55 and OR_*GA vs. AA*_=1.19). The TSA results indicated that the evidence of the effect was sufficient. In conclusion, *CTLA4* +49A/G polymorphisms increased the risk of T1D in children, and *CTLA4* +49A/G can be considered to be a genetic marker for T1D in children.

## INTRODUCTION

Type 1 diabetes (T1D) is the most common metabolic disorder in children, and it has serious short-term and long-term implications [1]. T1D increases dramatically in children aged 0-4 years [2], and more than 500,000 children developed T1D in 2015 (www.idf.org). The cause of T1D is not fully understood. In 1974, Nerup et al. [3] first identified the associations between human leukocyte antigen (HLA) variants and T1D. Recent studies have found that T1D clusters within families and twins [4, 5]. These studies have indicated that T1D is one of the most heritable types of common diseases.

T1D is an organ-specific autoimmune disease that results from the T cell-mediated destruction of pancreatic β cells [1]. The cytotoxic T-lymphocyte associated protein 4(CTLA-4) gene, which has been mapped to the insulin-dependent diabetes mellitus (IDDM) 12 locus (2q33), encodes a T-cell-specific transmembrane co-receptor [6]. The *CTLA-4* gene is known to contain multiple single nucleotide polymorphisms (SNPs): -318C/T in the promoter, +49A/G in exon 1, and a multiallelic dinucleotide repeat in the 3′untranslated region (UTR) of exon 4 [7]. The SNP of *CTLA-4* +49A/G locus (rs231775) has a gene dosage effect on the levels of sCTLA-4 [8]; therefore, *CTLA-4* +49A/G polymorphism has been widely studied in autoimmune diseases, and it has been reported to be involved in the development of Graves’ disease (GD) [9], systemic lupus erythematosus (SLE) [10], and diabetes mellitus [11]. Nistico et al. [12] first found a strong association between T1D risk and *CTLA-4* +49A/G polymorphisms in 1996, but this was not further confirmed by Yanagawa T et al.[13] in 1999. In addition, several meta-analysis studies [14–17] did not stratify by age, so the association between *CTLA-4* +49A/G polymorphisms and the risk of T1D in children is still not confirmed. Since children are a group that is relatively unaffected by confounders (e.g., environment and diet), the association between *CTLA-4* +49A/G polymorphisms and the risk of T1D in children can more realistically reflect genetic susceptibility to T1D.

Although Luo et al. [18] conducted a meta-analysis of this topic in 2012, that analysis did not include seven suitable studies [19–25]; moreover, recently other studies with adequate power have been published [26, 27]. Therefore, this present study conducted an updated systematic review and meta-analysis of studies describing the association between *CTLA-4* +49A/G polymorphisms and the risk of T1D in children. Furthermore, trial sequential analysis (TSA) was used to determine whether the currently available evidence was sufficient and conclusive.

## RESULTS

### Study characteristics

**Figure 1:**
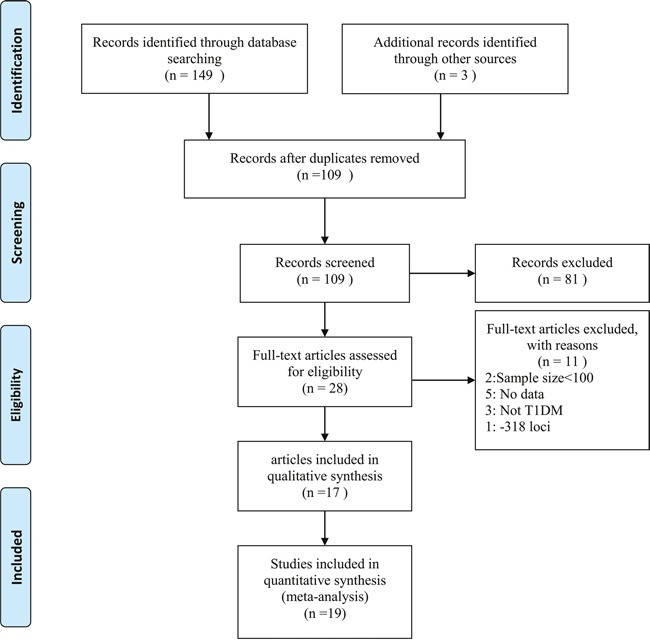
Flow diagram of included studies for this meta-analysis

Figure 1 shows the flow diagram of the literature selection process. A total of 17 articles were included for qualitative synthesis based on the inclusion and exclusion criteria, and two articles [21, 23] were further separated into four studies because they examined different racial groups (Table 1, S1 file); therefore, a total of 19 case-control studies, amounting to 3,797 cases and 3,981 controls, were finally pooled into the meta-analysis.

**Table 1:** Characteristics of the included studies

Study	Year	Country	Diagnosis criteria	Sample size		Age		Genotyping method	P* (HWE)
				Cases	Control	Cases	Control		
Lee	2000	China	The National Diabetes Data Group (1979)	253	91	7.1±3.7	adults	RFLP	0.378
Takara	2000	Japan	anti-GAD antibody (GAD65) and a urinary C-peptide level of <20 μg/day	51	107	10.9±7.7	-	RFLP	0.044
Cinek	2001	Czech	WHO glycaemic criteria	305	289	7.6±3.8	8.5±3.9	PCR-ARMS	0.458
Kamoun	2001	Tunisia	-	74	49	<15	-	RFLP	0.316
Kikuoka	2001	Japan	WHO Study Group(1985)	125	200	0.5-16	-	RFLP	0.287
Osei-Hyiaman	2001	China	The National Diabetes Data Group (1979)	350	420	0.3-15	0.3-15	SSCP	0.741
Osei-Hyiaman	2001	West African	The National Diabetes Data Group (1979)	182	201	0.3-15	0.3-15	SSCP	0.295
Mochizuki	2003	Japan	The Japan Diabetes Society and the American Diabetes Association	97	60	<16	matched	RFLP	0.539
Genc	2004	Turkish	the National Diabetes Data Group	48	80	13.45±0.47	11.93±0.91	RFLP	0.233
Dallos	2008	Slovak	Hyperglycemia and development of ketoacidosis	150	136	7.5± 4.0	matched	RFLP	0.079
Dallos	2008	Slovene	Hyperglycemia and development of ketoacidosis	170	95	9.7±4.3	matched	RFLP	0.579
Saleh	2008	Egypty	ketoacidosis or ketosis and continuous dependence on insulin	396	396	6.7±0.4	32.5±3.7	RFLP	0.501
Balic	2009	Chile	American Diabetes Association diagnostic criteria	300	310	10.8±4.1	11.0±2.2	RFLP	0.267
Jung	2009	Korea	WHO(1999)	176	90	7.5±4.0	-	RFLP	0.053
Momin	2009	Chile	the WHO diagnostic guidelines, clinical symptoms, absolute insulin-dependency, and pancreas-specific autoantibodies	261	300	9.47±3.32	10.66±3.05	RFLP	0.434
Douroudis	2009	Finnish	American Diabetes Association diagnostic criteria	404	725	9.8±3.4	-	RFLP	0.232
Perez	2009	Spanish	-	260	255	13.8±5.3	11.3±1.7	RFLP	0.115
Celmeli	2013	Turkish	WHO	91	99	11.7±5.1	9~30	RFLP	0.161
Mosaad	2013	Egypty	-	104	78	12.03±3.44	12.74±2.83	RFLP	0.01

Abbreviations: RFLP, restriction fragment length polymorphism; PCR-ARMS, polymerase chain reaction-amplification refractory mutation system; SSCP, polymerase chain reaction-single-strand conformation polymorphism; HWE, Hardy-Weinberg equilibrium.

Table 1 lists the characteristics of the included studies. Among the included studies, 13 case-control studies were conducted in Caucasian groups [8, 21–24, 26–32], and six in Asian groups [19–21, 25, 33, 34]. Most of the studies used the restriction fragment length polymorphism (RFLP) method for genotyping [8, 19, 20, 23–34]. Only one used a novel polymerase chain reaction-amplification refractory mutation system (PCR-ARMS) assay [22], and one used a polymerase chain reaction-single-strand conformation polymorphism (SSCP) assay [21]. Only two of the studies did not use HWE [26, 34].

### Risk of bias assessment

As described in Table 2, the answers for the ascertainment of the controls, population stratification and selective outcome report were “Yes” for all the included studies; therefore, the risk of bias was less likely. However, the risk of bias was highest in quality control for genotyping (68.4%, unclear for 13 studies). The other risk of bias comes from the diagnosis of T1D (26.3%, unclear for five studies), confound bias (31.6%, unclear for six studies), and HWE (10.5%, two studies out of HWE).

**Table 2:** Determination of risk assessment bias by included studies

Study	Ascertainment of T1D	Ascertainment of controls	Confounding Bias	Quality control for genotyping	HWE In control	Population stratification	Selective outcome report
Lee	Yes	Yes	Yes	Yes	Yes	Yes	Yes
Takara	Yes	Yes	Unclear	Unclear	No	Yes	Yes
Cinek	Yes	Yes	Yes	Yes	Yes	Yes	Yes
Kamoun	Unclear	Yes	Yes	Yes	Yes	Yes	Yes
Kikuoka	Yes	Yes	Yes	Unclear	Yes	Yes	Yes
Osei-Hyiaman	Unclear	Yes	Unclear	Unclear	Yes	Yes	Yes
Mochizuki	Yes	Yes	Yes	Unclear	Yes	Yes	Yes
Genc	Yes	Yes	Yes	Unclear	Yes	Yes	Yes
Dallos	Yes	Yes	Yes	Yes	Yes	Yes	Yes
Saleh	Yes	Yes	No	Yes	Yes	Yes	Yes
Balic	Yes	Yes	Yes	Unclear	Yes	Yes	Yes
Douroudis	Yes	Yes	Unclear	Unclear	Yes	Yes	Yes
Jung	Yes	Yes	Unclear	Unclear	Yes	Yes	Yes
Momin	Yes	Yes	Yes	Unclear	Yes	Yes	Yes
Perez	Unclear	Yes	Yes	Unclear	Yes	Yes	Yes
Celmeli	Yes	Yes	Yes	Unclear	Yes	Yes	Yes
Mosaad	Unclear	Yes	Unclear	Unclear	No	Yes	Yes

Abbreviations: T1D, Type 1 diabetes; HWE, Hardy-Weinberg equilibrium.

### Allele frequencies in different ethnicities

To assess the allele frequencies in different ethnicities, the allele frequencies in the controls were calculated based on the original data presented in the included studies. The G and A allele frequencies were 43.7% and 56.3%, respectively, in the Asian population, and 37.8% and 62.2%, respectively, in the Caucasian population. The GG, GA, and AA frequencies were 21.5%, 44.5%, and 34.1%, respectively, in the Asian population and 15.9%, 43.8%, and 40.3%, respectively, in the Caucasian population. A statistically significant difference was found between the two ethnicities (χ^2^=23.7, *P*<0.001 for allele frequency and χ^2^=22.7, *P*<0.001 for genotype frequency).

### Meta-analysis results

Table 3 shows the meta-analysis results of the association between *CTLA4* +49A/G polymorphisms and T1D risk. Significant heterogeneity was observed in the allelic model (*I*^2^=43.9 and *P*=0.001); therefore, a random effects model was used and the overall OR was 1.33 (95%CI=1.19-1.48). After the model was stratified by ethnicity, heterogeneity decreased significantly in the Asian population (*I*^2^=24.7 and *P*=0.249), but not in the Caucasian population (*I*^2^=53.4 and *P*=0.012). The ORs were 1.60 (95%CI=1.39-1.83) for the Asian population and 1.24 (95%CI=1.10-1.40) for the Caucasian population. These results did not significantly change after excluding the two studies that did not use HWE (Table 3).

**Table 3:** Associations between *CTLA4* +49A/G polymorphism and T1D risk in children

Comparison	Group	No. of studies	Test of association		*P*	Model	Heterogeneity	
			OR	95%CI			*I^2^%*	*P*
Allele G vs allele A	overall	19	1.33	1.19-1.48	<0.001	R	44.0	0.001
	Asian	6	1.60	1.39-1.83	<0.001	F	24.7	0.249
	Caucasian	13	1.24	1.10-1.40	0.001	R	53.4	0.012
	HWE	17	1.30	1.16-1.46	<0.001	R	60.9	0.001
	Age matched	11	1.24	1.06-1.46	0.008	R	67.1	0.001
GG vs GA	overall	19	1.35	1.18-1.54	<0.001	F	21.1	0.392
GG vs AA	overall	19	1.75	1.37-2.24	<0.001	R	57.5	0.001
	Asian	6	2.46	1.85-3.27	<0.001	F	39.7	0.141
	Caucasian	13	1.55	1.18-2.03	0.002	R	52.4	0.014
	RFLP	16	1.77	1.38-2.27	<0.001	R	45.6	0.024
	HWE	17	1.68	1.31-2.16	<0.001	R	58.8	0.001
	Age matched	11	1.51	1.07-2.13	0.019	R	64.0	0.002
GA vs AA	overall	19	1.26	1.09-1.46	0.002	R	40.4	0.036
	Asian	6	1.58	1.24-2.01	<0.001	F	37.6	0.156
	Caucasian	13	1.19	1.06-1.34	0.003	F	35.3	0.100
	RFLP	16	1.26	1.11-1.42	<0.001	F	26.9	0.153
	HWE	17	1.25	1.07-1.47	<0.001	R	46.0	0.020
	Age matched	11	1.21	1.01-1.45	0.039	R	44.8	0.053

Abbreviations: OR, odds ratio; CI, confidence intervals; HWE, Hardy-Weinberg equilibrium, RFLP, restriction fragment length polymorphism.

**Figure 2:**
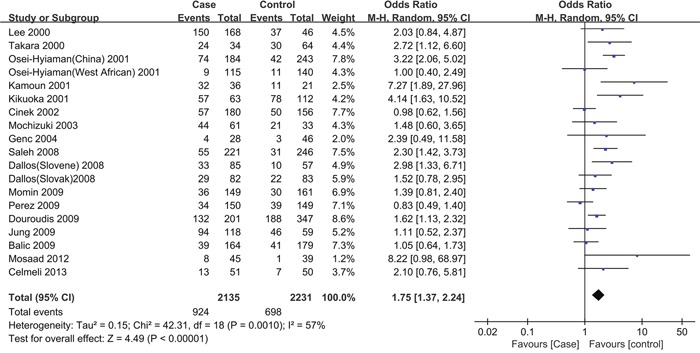
Forest plot for the association between CTLA-4 +49A/G polymorphisms and the risk of T1D in children (a codominant model: GG vs. AA) Using a random effect model. The squares and horizontal lines correspond to the studyspecific OR and 95 % CI. The diamond represents the summary OR and 95 % CI

**Figure 3:**
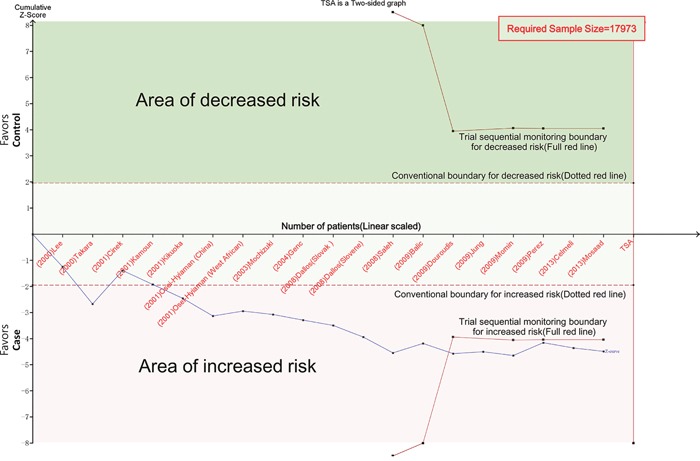
Trial sequential analysis for codominant model (GG vs. AA) The cumulative Z-curve (blue line) crossed the trial sequential monitoring boundary (red line), establishing sufficient and conclusive evidence. X-axis: the number of patients randomized; Y-axis: the cumulative Z-Score; α = 0.05 (two sided), β = 0.20 (power 80%), an anticipated relative risk reduction of 20%.

The overall ORs for GG vs. AA (OR_1_), GA vs. AA (OR_2_), and GG vs. GA (OR_3_) were 1.75, 1.26, and 1.35, respectively (Table 3). These findings indicate OR_1_>OR_2_>1 and OR_1_>OR_3_>1; therefore, a codominant model was suggested. Then, this meta-analysis estimated the gene effects for GG vs. AA and GA vs. AA. Significant heterogeneity was present for both OR_1_ (*I^2^*=57.5% and *P*=0.001) and OR_2_ (*I^2^*=40.4% and *P*=0.036). Thus, a random effects model was applied, and the overall gene effect was significant with a pooled OR_1_ and OR_2_ of 1.75 (95%CI=1.37-2.24, Figure 2) and 1.26 (95%CI=1.09-1.46), respectively. When using TSA, although the size of the samples in this meta-analysis did not reach the required sample size (17,973 for the GG vs. AA model, 12,558 for the GA vs. AA model), the cumulative Z-curve crossed the trial sequential monitoring boundary, establishing sufficient and conclusive evidence (Figure 3 and Supplementary Figure 1).

The results of the stratified analyses were not significantly different from the overall results (Table 3). The ORs for the Asian population (OR_*allelic model*_=1.60, OR_*GG vs. AA*_=2.46 and OR_*GA vs. AA*_=1.58) were higher than the ORs for the Caucasian population (OR_*allelic model*_==1.24, OR_*GG vs. AA*_=1.55 and OR_*GA vs. AA*_=1.19). The OR value decreased in the age-matched subgroup in comparison to the pooled results.

### Heterogeneity analysis

**Figure 4:**
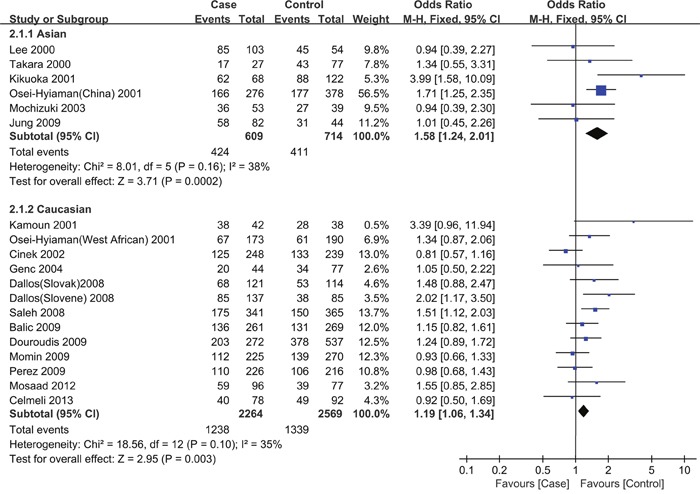
Forest plot for the association between CTLA-4 +49A/G polymorphisms and the risk of T1D in children stratified by ethnicity (a codominant model: GA vs. AA) Using a fixed effect model. The squares and horizontal lines correspond to the studyspecific OR and 95 % CI. The diamond represents the summary OR and 95 % CI

An exploration of the sources of heterogeneity was performed using a subgroup analysis stratified by ethnicity, genotyping method, and HWE. As shown in Table 3 and Figure 4, a comparison of GA vs. AA revealed that heterogeneity was significantly reduced in the subgroup analysis stratified by ethnicity and genotyping method. This suggests that ethnicity and the genotyping method are the main source of heterogeneity. The pooled OR value did not change significantly in the heterogeneity analysis.

### Sensitivity analysis

Two studies that did not apply the HWE principle were first excluded in the sensitivity analysis, and this did not change the significance of all the ORs (Table 3). Two studies with different genotyping methods were further excluded, as shown in Table 3 (the subgroup analysis for the RFLP group), and the recalculated pooled OR did not change significantly. Finally, a sensitivity analysis was performed by excluding each study, one-by-one, and no statistically significant changes in the ORs were observed (data not shown).

### Publication bias

**Figure 5:**
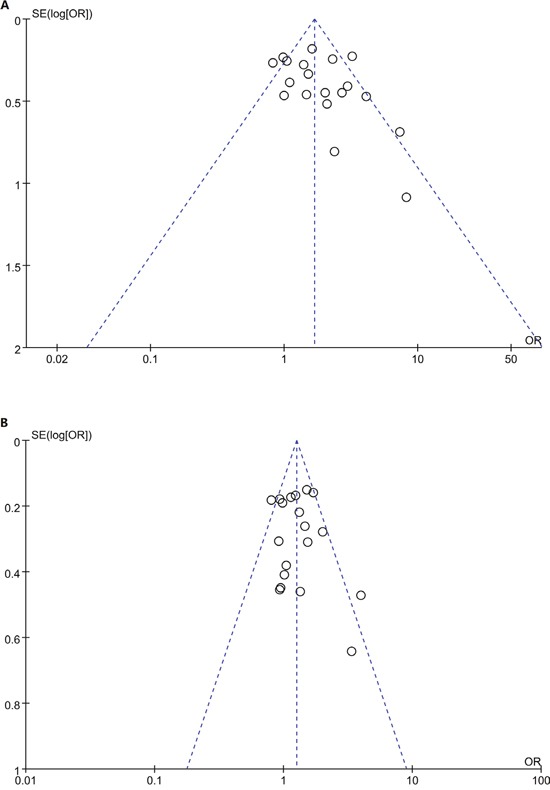
Funnel plot for studies of the association between CTLA4 +49A/G polymorphism and T1D risk in children in codominat model A. GG vs. AA; B GA vs. AA. The horizontal and vertical axes correspond to the OR and 95% CI.

Figure 5 shows the funnel plots for GG vs. AA and GA vs. AA. The two funnel plots were symmetrical. Begg’s funnel plot and Egger’s test were also conducted, and the statistical results showed evidence of funnel plot symmetry (*P_Egger’s_*_test_>0.05).

## DISCUSSION

In this study, we found that children carrying the G allele in *CTLA4* +49A/G had a 1.33-fold increased risk of developing T1D in comparison to children carrying the A allele; children who had a GG or GA genotype had a 1.75- or 1.26-fold higher risk, respectively, of developing T1D than children who had the AA genotype. These risk effects were further confirmed by TSA.

Hamzeh et al. [35] found that the alleles *HLA-DQB1* *02:01 and *03:02 and the haplotypes DR3 and DR4 were significant risk factors for T1D. In contrast, HLA-DQA1*01:01, DQB1*05:03, *06:02, *06:03, and *06:04 were protective factors against T1D. It has been found that *HLA-DQ-* and *HLA-DRB1*-containing genotypes contribute to about 50% of the risk associated with developing T1D [36, 37]. Other genes, such as insulin, protein tyrosine phosphatase, non-receptor type 22 (*PTPN22*), and interleukin 2 receptor alpha (*IL2RA*), are also candidate genes that are associated with the risk of T1D [6]. Hence, current evidence supports the hypothesis that T1D is due to interactions between the environment and genetic factors, and genetic variation has a clinically important impact on the risk of T1D. Considering the sufficient evidence from the TSA results, we recommend *CTLA4* +49A/G as a genetic marker to assess the T1D risk for children in clinical contexts.

Our results were partly consistent with a meta-analysis performed by Luo et al. [20] in 2012 (OR=1.21, 95%CI=1.09-1.33 for the G vs. A allele; OR=1.42, 95%CI=1.16-1.75 for the GG vs. AA genotype). In contrast, we further observed a 1.26-fold higher risk for children who had the GA genotype in comparison to those carrying the AA genotype. The inconsistent results may be due to the larger number of studies and the larger sample size in our meta-analysis (19 studies vs. 10 studies [18], and 7,778 vs. 2,422 subjects [18]). In addition, we also applied TSA to estimate the threshold for statistical significance according to the quantified strength of the evidence. The TSA results showed insufficient evidence of the effect, and they were potentially spurious in the meta-analysis performed by Luo et al. (Supplementary Figure 2. GG vs. AA); in contrast, our study established sufficient and conclusive evidence. Another difference between our study and previous meta-analysis [18] was that we used the best genetic model to avoid the problem of multiple comparisons. Therefore, we believe that our results are more robust than previous findings.

The development of T1D in children has been shown to be caused by different genetic susceptibility factors between different populations [6]. Indeed, the incidence of T1D in children varies geographically—e.g., it is lowest in China and Venezuela and highest in Finland and Sardinia; moreover, the trends estimated for continents showed a 4.0% increase in T1D in Asia, which is higher than the increase in Europe (3.2%) [38]. Chen et al. [16] found a wide variation in the risk allele frequency of the *CTLA4* +49A/G polymorphism among the controls across different ethnicities. Our study also observed significant difference in the allele and genotypes frequencies in *CTLA4* +49A/G polymorphisms between Asian and Caucasian children. Hence, we also conducted a subgroup analysis stratified by ethnicity. The association between *CTLA-4* +49A/G polymorphisms and the risk of T1D was greater in Asian children than in Caucasian children, which is consistent with the results of a meta-analysis of all ages [14]. However, in a meta-analysis of all ages from 2005, Kavvoura et al. [15] reported that the risk of T1D associated with *CTLA-4* +49A/G polymorphisms was higher in Caucasians than in Asians. These inconsistent results are understandable because these meta-analyses reviewed different studies with different sample sizes and different inclusion and exclusion criteria. Moreover, other basic characteristics, such as gender and lifestyle, should be considered when explaining the differences.

It should be noted that this present study examined significant heterogeneity. Therefore, we performed a subgroup analysis of ethnicity, HWE, and genotyping methods, and the differences between the case studies and the control studies. As expected, heterogeneity decreased significantly in the subgroup stratified by ethnicity, which not only strongly confirms that different races are the main source of heterogeneity in this meta-analysis, but also indicates the different degree of risk of T1D associated with *CTLA4* +49A/G polymorphisms in different ethnicities. Therefore, because only Asian and Caucasian children were studied in this meta-analysis, further studies on other races are needed.

Our meta-analysis has several limitations. First, we detected substantial heterogeneity between the examined studies, which would influence the reliability of our conclusions, even if the corresponding pooled OR value was not significantly changed in the sensitivity analysis. Second, we restricted the research to English publications. In addition, one of the included studies focused on West African children. Therefore, potential publication bias should be noted, although a Begg’s funnel plot and an Egger’s test showed no publication bias. Third, the implementation of some of the studies was reported poorly. As shown in Table 2 and Table 3, some studies selected healthy adults as controls, and some studies did not apply the HWE principle, which could lead to false-negative or false-positive results. Fortunately, the results were not significantly changed after we excluded those studies. Finally, since approximately 50% of the genetic risk of T1D is conferred by HLA genotypes [39], theoretically, the normalization procedure used in our meta-analysis should remove any influence that HLA genotypes could have on the risk of T1D in children. However, few studies have examined *HLA* and *CTLA4* together; thus, this should be investigated in future studies.

Despite these limitations, the present study has some advantages in comparison to previous studies. First, we used TSA to determine the association between the risk of childhood T1D and *CTLA4* +49A/G polymorphisms. The TSA results demonstrate that the current evidence is sufficient, thereby suggesting that our results are reliable. Second, this study has more statistical power than previous meta-analyses, and we also selected the best genetic model to avoid multiple comparisons. Third, our study was performed using PRISMA guidelines and the recommendations of the Cochrane Collaboration, although our study was not registered with any specific protocol. In summary, our study is the most recent and the most comprehensive meta-analysis of this topic to date. *CTLA4* +49A/G can be considered to be a genetic marker for T1D in children.

## MATERIALS AND METHODS

This study was performed according to the Preferred Reporting Items for Systematic Reviews and Meta-analyses (PRISMA) 2009 statement [40](Supplementary Table 1). There was no registered protocol.

### Search strategy

Two investigators (Bo Wang and Wei Du) conducted a systematic literature search of the EMBASE, PubMed, and Cochrane Library databases for papers related to the studied topic that were published up to March 30, 2016. The following search terms were used: *T1DM* or *T1D* or *type 1 diabetes* or *type 1 diabetes mellitus* in combination with *polymorphism* or *polymorphisms* in combination with *CTLA4* or *Cytotoxic T-Lymphocyte Associated Antigen-4* or *rs231775* in combination with *children* or *child* or *childhood*. In addition, reference lists in the relevant publications were also hand-searched to identify additional records. Only English publications were searched.

### Selection criteria

Two reviewers (Bo Wang and Wei Du) identified eligible articles based on a full-text review. To be included, studies had to be case-control or cohort studies describing the association between *CTLA4* +49A/G polymorphisms and T1D risk in children (≤16 years); moreover, the genotype frequencies of *CTLA4* +49A/G loci in the cases and controls could be extracted from these studies (or could be obtained after contacting the authors).

Exclusion criteria were: studies with a sample size <100 for limited statistic power; in addition, family-based studies, review articles, letters, case reports, editorials, and conference abstracts were also excluded. Any disagreement was resolved by consensus.

### Data extraction

Two investigators (Bo Wang and Wei Du) independently extracted data from the included studies, and it was critical that the final data extracted by the two investigators be the same. Disagreements were resolved by discussion and consensus. The data included: the first author’s name, year of publication, the country and ethnicity of the cases, the age of the patients and healthy controls, the sample size, the diagnosis criteria, the genotyping method, and the genotype frequencies of the cases and controls. Hardy-Weinberg equilibrium (HWE) was calculated from the extracted data. A single article with different populations was further categorized as different studies. If the literature did not provide sufficient data, the investigators tried to contact the author by email to obtain the original data.

### Risk of bias assessment

To assess the quality of each study, we used the widely-used quality assessment criteria for genetic association studies defined by Thakkinstian et al. [41], with some of the criteria modified according to the study by Dong et al.[42](Supplementary Table 2). The score consists of the following factors: ascertainment of diagnosis of T1D and controls, quality control for genotyping, population stratification, confounding bias, selective reporting (for replication studies), and HWE in the control group. Each factor was rated using the following question: Is there a low risk of bias? If the answer was “Yes”, a low risk of bias was indicated; if the answer was “No” a high risk, of bias was indicated. If the answer was “Unclear”, insufficient information was available for the assessment.

### Statistical analysis

In this study, unadjusted odds ratios (ORs) and the corresponding 95% confidence intervals (CIs) were used to measure the strength of the association between *CTLA4* gene +49A/G polymorphisms and T1D risk in children. Previous studies have indicated that G is the risk allele; therefore, we first calculated the ORs of GG vs. AA (OR_1_), GA vs. AA (OR_2_), and GG vs. GA (OR_3_), and we then selected the best genetic model based on the meta-analysis method used in molecular association studies [43].

To assess for the heterogeneity of the ORs across studies, the Cochrane Q statistic and I-squared (*I^2^*) statistic were calculated, and a fixed effects model was used if *I^2^*<50% and *P*≥0.1, whereas a random effects model was used if *I^2^*≥50% and *P*<0.1 [44, 45]. The HWE in each study was assessed using the chi-square test for goodness of fit only in the control groups, and a P-value <0.05 was considered to be out of HWE. If significant heterogeneity was found in a compared genetic model, a subgroup analysis of ethnicity, genotyping methods, and HWE was conducted.

The stability of the results was assessed using sensitivity analysis. This method omits a study, one-by-one, and then recalculates the pooled ORs. If the ORs did not change significantly, the results were considered robust. The possibility of publication bias was assessed using Begg’s funnel plot and Egger’s test [46, 47].

Analyses were performed using STATA software (version 12.0; Stata Corporation, College Station, Texas, USA) and RevMan 5.3 (Cochrane Collaboration).

### Trial sequential analysis

Meta-analyses may result in type I errors due to random error from the studies included in the meta-analysis that had a small sample size, publication bias, and low quality, and studies whose conclusions tended to be changed by later studies with a larger sample size [48]. TSA can overcome these limitations and further reveal insufficient information size and potentially false positive results in a meta-analysis [48, 49]. Therefore, we performed TSA according to an overall 5% risk of a type I error, a power of 80%, and relative risk reduction of 20%. TSA software version 0.9 beta (http://www.ctu.dk/tsa) was used in this study. When the cumulative Z-curve crosses the trial sequential monitoring boundary or enters the futility area, a sufficient level of evidence may have been reached, and no further trials are needed. If the cumulative Z-curve does not cross any of the boundaries, and the required information size has not been reached, there is insufficient evidence to reach a conclusion [50].

## SUPPLEMENTARY MATERIALS FIGURES AND TABLES




