# Supplementary material for: Cytotoxic T-lymphocyte-associated protein 4 +49A/G polymorphisms contribute to the risk of type 1 diabetes in children: An updated systematic review and meta-analysis with trial sequential analysis
Source: Oncotarget. 2017 Jan 2;8(6):10553–64. doi: 10.18632/oncotarget.14457 (PMC5354680; doi:10.18632/oncotarget.14457)
Supplement: Supplementary file 1 [file oncotarget-08-10553-s001.pdf]

Cytotoxic T-lymphocyte-associated protein 4 +49A/G polymorphisms contribute to the risk of type 1 diabetes in children: An updated systematic review and meta-analysis with trial sequential analysis

SUPPLEMENTARY DATA

Search strategy

(((((((T1DM) OR type 1 diabetes) OR T1D) OR type 1 diabetes mellitus)) AND ((polymorphism) OR polymorphisms)) AND (((CTLA4) OR Cytotoxic T-Lymphocyte Associated Antigen-4) OR rs231775)) AND (((children) OR child) OR childhood).

2. Additional records identified through other sources [1-3]

3. Full-text articles excluded, with reasons

Sample size<50:[4, 5]

No data:[6-10]

No T1DM:[11-13]

-318 loci:[14]

1. Cinek O, Drevinek P, Sumnik Z, Bendlova B, Kolouskova S, Snajderova M, Vavrinec J: The CTLA4 +49 A/G dimorphism is not associated with type 1 diabetes in Czech children. European journal of immunogenetics: official journal of the British Society for Histocompatibility and Immunogenetics 2002, 29:219-222.
2. Pérez B F, Codner D E, Angel B B, Balic N I, Carrasco P E: +49 A/G genetic polymorphism of cytotoxic T lymphocyte associated antigen 4 (CTLA-4) in type 1 diabetes: Association with autoantibodies and cytokines. Revista medica de Chile 2009, 137:321-328.
3. Saleh HM, Rohowsky N, Leski M: The CTLA4 -819 C/T and +49 A/G dimorphisms are associated with Type 1 diabetes in Egyptian children. Indian journal of human genetics 2008, 14:92-98.
4. Arafa RM, Desouky SM, Emam SM, Abed NT, Mohamed SY: Detection of Cytotoxic T-Lymphocyte Associated Antigen-4 Gene Polymorphism in Type 1 Diabetes Mellitus. The Egyptian journal of immunology / Egyptian Association of Immunologists 2015, 22:49-57.
5. Pruul K, Kisand K, Alnek K, Metskula K, Heilman K, Peet A, Varik K, Uibo R: Expression of B7 and CD28 family genes in newly diagnosed type 1 diabetes. Human immunology 2013, 74:1251-1257.
6. Bassuny WM, Ihara K, Matsuura N, Ahmed S, Kohno H, Kuromaru R, Miyako K, Hara T: Association study of the NRAMP1 gene promoter polymorphism and early-onset type 1 diabetes. Immunogenetics 2002, 54:282-285.
7. Lempainen J, Vaarala O, Makela M, Veijola R, Simell O, Knip M, Hermann R, Ilonen J: Interplay between PTPN22 C1858T polymorphism and cow's milk formula exposure in type 1 diabetes. Journal of autoimmunity 2009, 33:155-164.
8. Mlynarski W, Zmyslowska A, Kubryn I, Perenc M, Bodalski J: [Factors involved in ketoacidosis at the onset of type 1 diabetes in childhood]. Endokrynologia, diabetologia i choroby przemiany materii wieku rozwojowego: organ Polskiego Towarzystwa Endokrynologow Dzieciecych 2003, 9:23-28.
9. Steck AK, Bugawan TL, Valdes AM, Emery LM, Blair A, Norris JM, Redondo MJ, Babu SR, Erlich HA, Eisenbarth GS et al: Association of non-HLA genes with type 1 diabetes autoimmunity. Diabetes 2005, 54:2482-2486.
10. Valdes AM, Varney MD, Erlich HA, Noble JA: Receiver operating characteristic analysis of HLA, CTLA4, and insulin genotypes for type 1 diabetes. Diabetes care 2013, 36:2504-2507.
11. Jonson CO, Lernmark A, Ludvigsson J, Rutledge EA, Hinkkanen A, Faresjo M: The importance of CTLA-4 polymorphism and human leukocyte antigen genotype for the induction of diabetes-associated cytokine response in healthy school children. Pediatric diabetes 2007, 8:185-192.
12. Lin JJ, Lin KL, Wang Y, Wang HS, Hsia SH, Chang LY: CTLA-4+49 A/G polymorphism and antiglutamic acid decarboxylase antibody-associated encephalopathy in Taiwanese children. Brain & development 2016, 38:419-426.
13. Wallden J, Ilonen J, Roivainen M, Ludvigsson J, Vaarala O: Effect of HLA genotype or CTLA-4 polymorphism on cytokine response in healthy children. Scandinavian journal of immunology 2008, 68:345-350.
14. Lee YJ, Lo FS, Shu SG, Wang CH, Huang CY, Liu HF, Wu CC, Yang TY, Chang JG: The promoter region of the CTLA4 gene is associated with type 1 diabetes mellitus. Journal of pediatric endocrinology & metabolism: JPEM 2001, 14:383-388.

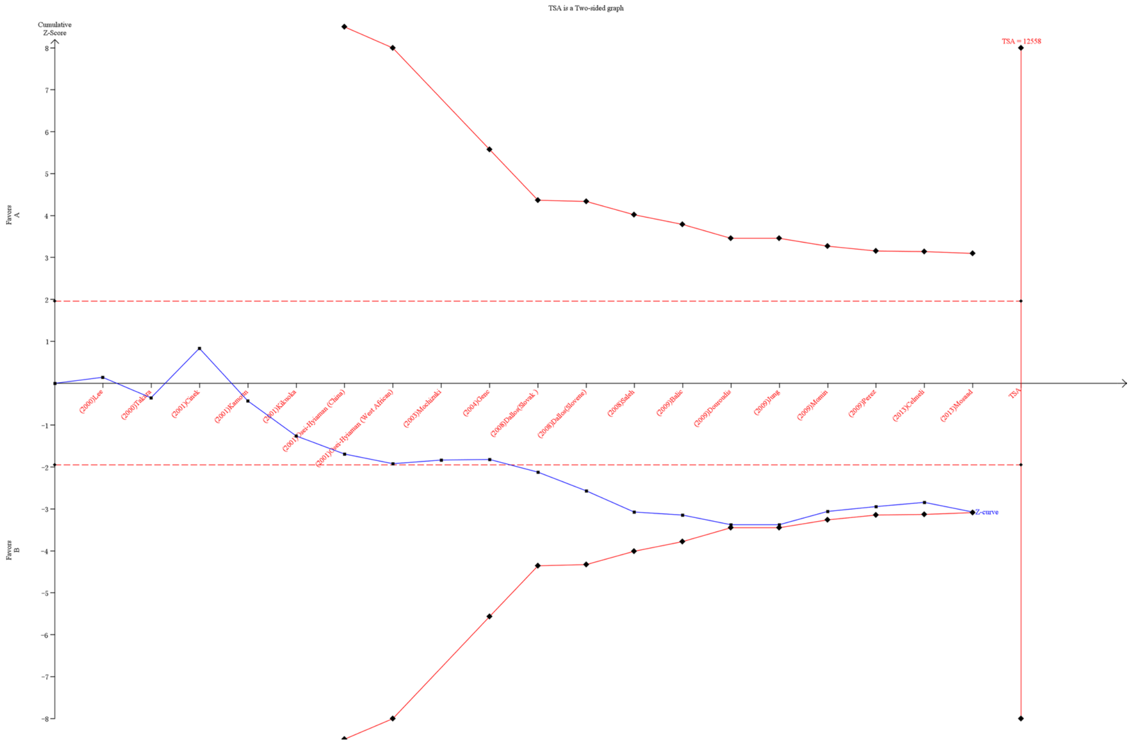

Supplementary Figure 1: Trial sequential analysis for codominant model (GA vs. AA).

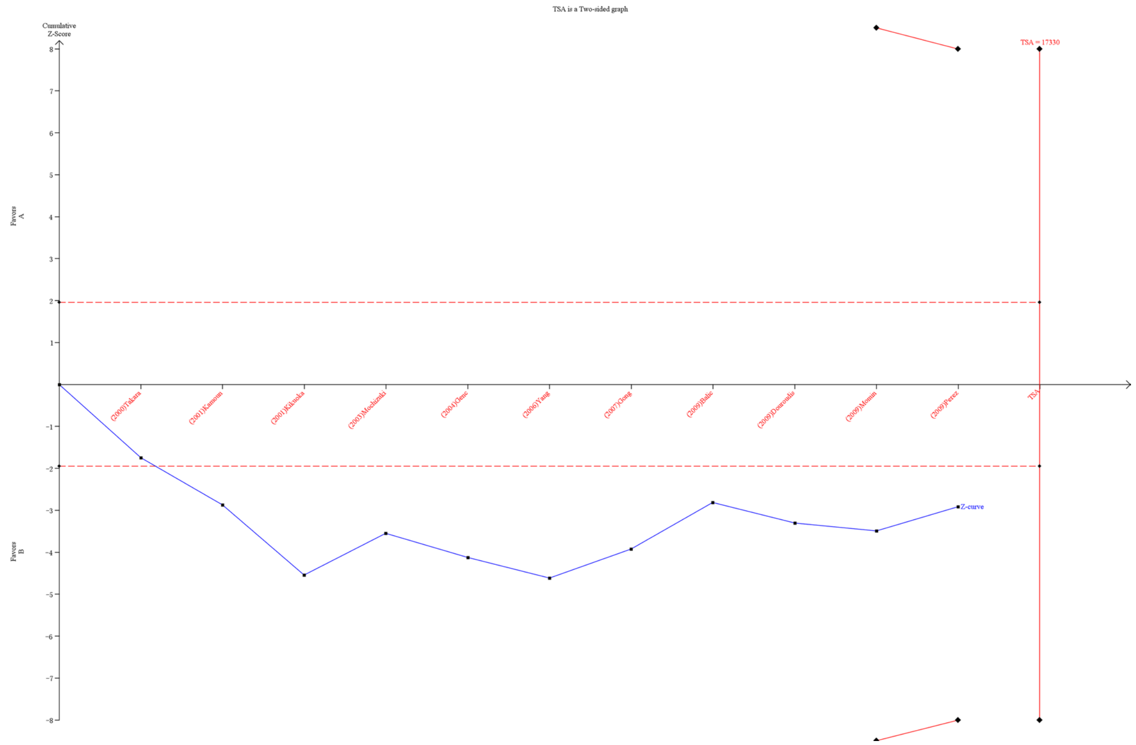

Supplementary Figure 2: Trial sequential analysis for codominant model in a previous study by Luo et al. (GG vs. AA).

Supplementary Table 1: PRISMA 2009 Checklist

See Supplementary File 1

Supplementary Table 2: Risk of bias assessment

Domain and item	Low risk of bias
Ascertainment of T1D	
Clearly described objective criteria of diagnosis of T1D	Yes
Not clearly described	No
Did not mention	Unclear
Ascertainment of controls	
Controls were non-T1D and without family history	Yes
Mentioned the sources of controls	Yes
Not described	No
Quality control for genotyping	
Quality control for genotyping was performed together	Yes
Using different genotyping methods to confirm the results	Yes
Using Marker	Yes
Did not mention	Unclear
Population stratification	
No difference in ethnic origin between cases and controls	Yes
Use of controls who were not related to cases with clearly identification	Yes
Use of some controls who came from the same family	No
No report of what was done	Unclear
Confounding bias	
Controls for confounding variables (e.g., age or gender) in analysis	Yes
Not controlled for confounding variables	No
Not mentioned	Unclear
Selective reporting (for replication studies)	
Reported results of all polymorphisms mentioned in the objectives, no significant or not	Yes
Reported results of only significant polymorphisms	No
HWE in the control group	
Fall in HWE	Yes
Out of HWE	No

T1D, type 1 diabetes; HWE, Hardy-Weinberg equilibrium.
